# Effect of Adaptive, Rotary, and Manual Root Canal Instrumentation in Primary Molars: A Triple-Armed, Randomized Controlled Clinical Trial

**DOI:** 10.3390/biology10010042

**Published:** 2021-01-10

**Authors:** Bhaggyashri A. Pawar, Ajinkya M. Pawar, Anuj Bhardwaj, Dian Agustin Wahjuningrum, Amelia Kristanti Rahardjo, Alexander Maniangat Luke, Zvi Metzger, Anda Kfir

**Affiliations:** 1 Department of Oral Health and Advanced Dentistry, Sir H N Reliance Foundation Hospital and Research Center, Mumbai 400004, Maharashtra, India; bhaggyashripawaar@gmail.com; 2 Department of Conservative Dentistry and Endodontics, Nair Hospital Dental College, Mumbai 400034, Maharashtra, India; ajinkya@drpawars.com; 3 Department of Conservative Dentistry, Faculty of Dental Medicine, Universitas Airlangga, Surabaya City, East Java 60132, Indonesia; dranuj_84@yahoo.co.in (A.B.); ameliakristanti94@yahoo.com (A.K.R.); 4 Department of Clinical Sciences, Ajman University, College of Dentistry, Ajman 346, UAE; a.luke@ajman.ac.ae; 5 Department of Endodontology, The Goldschleger School of Dental Medicine, Tel Aviv University, Tel Aviv 69978, Israel; metzger@post.tau.ac.il (Z.M.); andak@post.tau.ac.il (A.K.)

**Keywords:** anatomic instrumentation, hand files, Kedo-S, pediatric rotary files, primary molars, XP-endo Shaper

## Abstract

**Simple Summary:**

Untimely loss of primary molars may lead to undesirable consequences; hence, pulpectomy is considered a rational treatment approach to avoid it. Advances in root canal instrumentation by means of motorized files while treating primary teeth have reduced the chair-side time and have also exhibited better quality of obturation. This helps reduce the patient’s anxiety about pulpectomy procedures. However, root canals in primary teeth exhibit a larger perimeter and are irregular in shape, posing a challenge to currently used motorized endodontic files. Instrumenting root canals in three dimensions is the need of the hour and is vastly explored in the literature, albeit for adult dentition. The application of adaptive root canal instrumentation for pulpectomies in primary teeth is yet to be reported in the literature.

**Abstract:**

This clinical trial focused on collating the instrumentation time and quality of root canal obturation in primary molars treated with three instrumentation techniques: adaptive, rotary, and manual. A triple-armed, randomized controlled clinical trial was performed on 75 primary molars requiring pulpectomy treatment, divided into three groups (*n* = 25 per group). The teeth in Group 1 were instrumented with an adaptive technique (XP-endo Shaper, FKG Dentaire, La Chaux-de-Fonds, Switzerland), Group 2 with pediatric rotary files (Kedo-S; D1 and E1), and Group 3 with a manual technique (hand K-files). The apical size of the final instrumentation was maintained at #30 for all groups. Instrumentation time and the grade of the root canal obturation were evaluated. Instrumentation duration was recorded, employing a digital stopwatch from the insertion of the first file until the completion of final irrigation. Obturation quality was assessed using radiographs. The criteria taken as a reference for obturation were: optimal (1 mm short of the apex), underfilled (2 mm short of the apex), or overfilled (beyond the apex). The use of an adaptive technique was associated with the lowest instrumentation time (*p* < 0.0001) when used for instrumenting primary molars and with the highest root canal filling quality of the three groups. The application of the new concept of adaptive instrumentation for pulpectomy of primary molars was a favorable technique, considering the significant reduction in instrumentation time and better obturation.

## 1. Introduction

A current trend in pediatric dentistry is to perform pulpectomy procedures on necrotic primary teeth to maintain their function and avoid extraction and space loss [1]. The aim of root canal instrumentation in primary teeth is the mechanical shaping of the entire root canal space, chemical cleansing (using a desired root canal irrigant to the apex), and subsequent obturation (to the apex), which must all take place in a short duration of time [2,3]. Root canal instrumentation should result in the removal of vital and necrotic pulp tissue, infected dentine, and debris from the root canal system and its irregularities [2,3].

Conventionally, pulpectomy procedures were carried out using hand instrumentation (reamers, K-files, or H-files). Barr et al. introduced the use of rotary file instrumentation for this procedure, and the motorized instrumentation was found to be an efficient technique [4]. With the use of rotary files, instrumentation time decreased, increasing children’s cooperation during treatment [5]. In spite of the fact that rotary files have been widely used for root canal treatments of permanent teeth, their use for primary dentition is still emerging. 

Nickel-titanium (NiTi) rotary instruments are quick, safer, and precision-aligned, with a minimized risk of procedural errors. They also provide a good taper, thus aiding to achieve better obturation quality [6]. Several rotary files have been introduced and clinically evaluated for use in pediatric endodontics [7,8,9,10,11,12,13]. Rotary files have a solid central metal core with a rotating blade and flutes. Considering that canals are simple, straight, and narrow, with a round cross-section, these endodontic files may effectively achieve the goals of root canal shaping. However, in cases where the root canal is large and/or irregularly shaped, these files may become inadequate. They may leave most of the root canal space unaltered, thereby potentially leaving behind infected tissue [14,15]. Primary molars often exhibit ovoid- or ribbon-shaped root canals in their coronal part and oval shapes toward the apex [16]. Such canals constitute a crucial challenge for adequate root canal cleaning, shaping, and disinfection, chiefly when rotary instruments are used for shaping [17].

**Figure 1:**
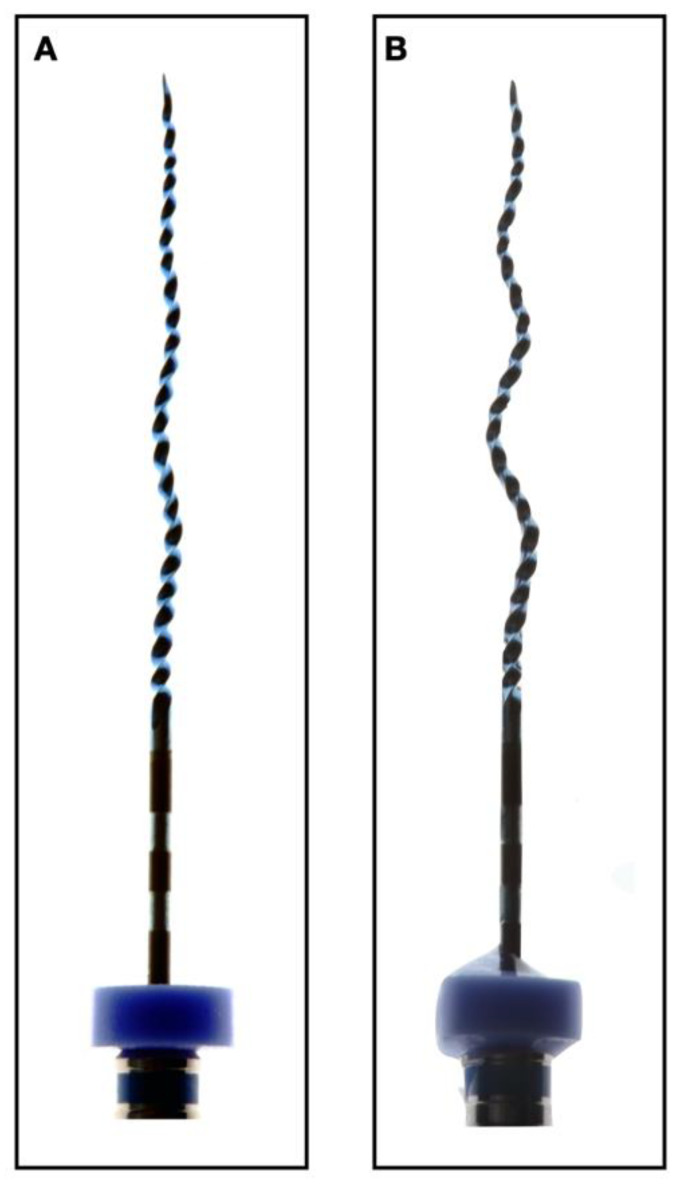
Temperature-dependent shape transition of the XP-endo Shaper. (**A**) XP-endo Shaper file at room temperature (20 °C); (**B**) the same file at body temperature (35 °C). The extremely flexible file will push itself into canal recesses not reachable by rotary NiTi files.

Recently, a new concept of an adaptive mechanized file was developed: the snake-shaped XP-endo Shaper file (FKG Dentaire, La Chaux-de-Fonds, Switzerland). It is manufactured from an innovative, thermomechanically treated NiTi alloy (Max-Wire, Martensite-Austenite Electropolished File). The wire has a size/taper of 30/0.01, which, according to the manufacturer, makes it more flexible and resistant to cyclic fatigue. Its booster tip features six cutting edges and a smooth transition from the base of the tip to the helical shaft [18]. The shape of this snake-shaped file is temperature-dependent; it is relatively straight at room temperature (M-phase) and transforms to a more “snake-like” shape when exposed to root canal temperature (A-phase) (Figure 1). When rotated at body temperature, the “envelope of motion” of the file results in a final canal preparation of a minimum of 30/0.04. These features enable the instrument to enlarge a canal from an apical size of #15 to #30 with a single instrument, without the need for long-established incremental instrument sizes. 

The file was specifically designed to adapt to the 3D morphology of root canal systems, including those with an oval cross-section. The file’s “envelope of motion” is flexible and may contract and expand as it is moved along the length of the root canal, which results in asymmetrical motion. This asymmetrical motion of the file results in reaching canal irregularities and addressing most of the root canal space [19]. It has been reported as being more effective compared to solid metal-cored rotary files when used in nonround root canals [20].

To the best of the authors’ knowledge, there is no report on the use of this adaptive endodontic instrumentation technology in primary dentition to date. Thus, it was important to a conduct systematic clinical trial to arrive at a definitive and conclusive outcome for this approach. The current clinical study aimed to respond to this gap in knowledge and compare the instrumentation time and obturation quality of the XP-endo Shaper adaptive system with those of the pediatric rotary file Kedo-S and the manual hand K-files instrumentation serving as the control.

## 2. Materials and Methods

The current investigation consisted of a triple-armed, single-blinded, randomized clinical trial following Ethical Committee approval from the College of Dental Sciences and Hospital, Rau, Indore, India (CDSH/IEC/2018-19/004. The sample size was determined, referring to a previous trial reported by Priyadarshini et al. [21], where instrumentation time and obturation quality were assessed. The power was set at 80%, with alpha-level (α) set at 0.05. The means of instrumentation times in minutes (min) from the previous studies were 3.4827 ± 0.48657 and 6.2167 ± 0.30978 for the Kedo-S and hand files, respectively. The allocation ratio used was 1:1. With the above parameters, the target sample size was derived to 15 samples per group, but we increased it to 25 samples per group, 75 patients total for the current randomized clinical trial.

### 2.1. Patient Recruitment and Allocation 

**Figure 2:**
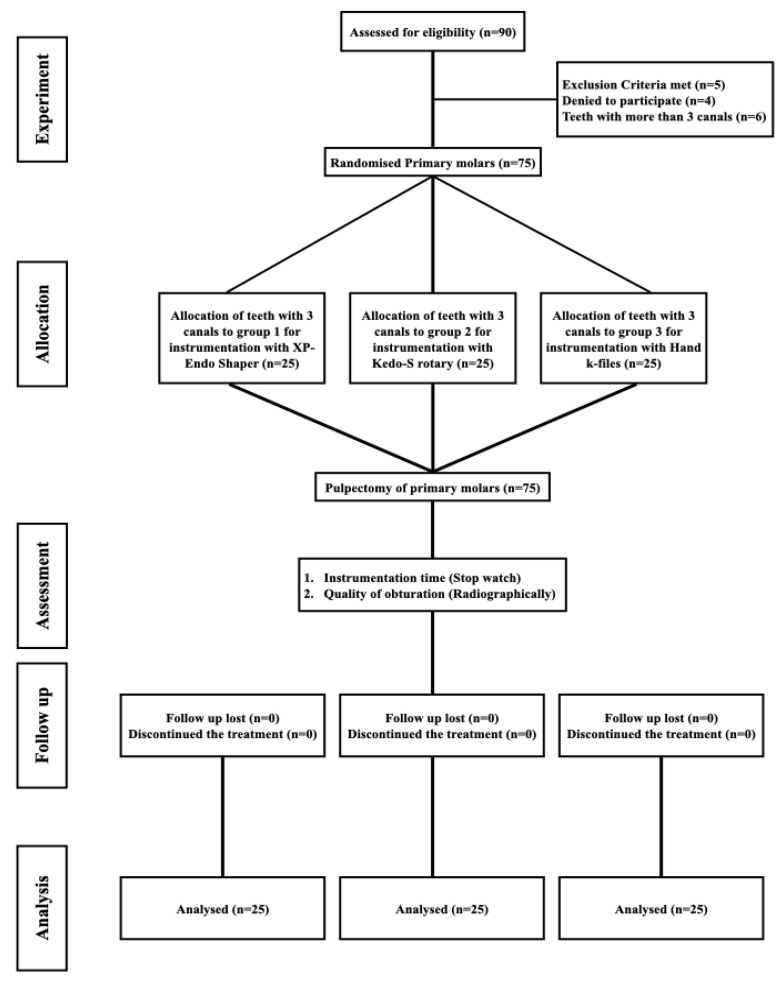
CONSORT flow chart complied during the different stages of this triple-armed, randomized clinical trial.

The current study was executed following the guidelines of the Consolidated Standards of Reporting Trials (CONSORT) group for planning and reporting clinical trials (Figure 2). Patients in need of pulpectomy in primary molars (first and second, maxillary and mandibular) were included in the current study. A total of 90 patients were recruited after obtaining informed consent from the parents. Patients of both genders and betwixt the age group of 4 and 9 years were included in the study.

The inclusion criteria were patients with necrotic posterior teeth containing at least one necrotic pulp canal, abscess, or sinus tract or radiolucency in the furcation or periapical area and presenting a behavior rating of 4 (+) or 5 (++), according to the modified Frankl scale of behavior assessment proposed by Wright [22]. Only teeth with a minimum of 2/3 root structures remaining (confirmed radiographically) and ample crown structure for rubber dam clamp placement and crown placement were included. After access cavity, teeth with 3 canals were included in the present study for the standardization of instrumentation time, treating the same number of canals for the 3 groups.

The exclusion criteria were patients who failed to provide informed consent, presenting a Frankl scale of behavior assessment of less than 4, with any history of systemic illness, nonrestorable teeth, perforated pulpal floor, imprudent mobility, or pathological root resorption, as well as teeth with more than 3 canal orifices. 

Following the abovementioned criteria, a total of 75 patients were recruited for the study, which included the following teeth; #54 (*n* = 12), #55 (09), #64 (12), #65 (*n* = 12), #74 (*n* = 12), #75 (*n* = 3), #84 (*n* = 12), and #85 (*n* = 3). Each set of primary molars was assigned sequential numbers in the order in which they were enrolled and divided into 3 groups of treatment (*n =* 25 per group) for the root canal instrumentation of primary molar teeth, using adaptive XP-endo Shaper rotary files, Kedo-S pediatric rotary files, and hand K-files, according to computer-generated randomization that was prepared before starting the study. Each group consisted of the same number of each type of maxillary and mandibular first and second primary molars for standardization. 

All root canal treatments were accomplished in a single visit by the same operator. In the present study, the evaluating observer and the analyst were blinded to the treatment protocol for the three groups. However, as the instrumentation files have recognizable characteristics, they were potentially unable to be blinded from the operator who performed the root canal treatment.

### 2.2. Root Canal Instrumentation

Pulpectomy was performed on the selected molars in a single visit, after administration of local anesthesia (XICAINE 2% adrenaline I.P. 1:80,000, ICPA Health Products Ltd., Mumbai, India). Following the subjective and objective signs of the local anesthesia, rubber dam (GDC Marketing, Hoshiarpur, Punjab, India) isolation was performed. The decayed substance was excavated, and an access opening was gained employing a #4 round carbide bur (Dentsply Maillefer, Tulsa, OK, USA) at elevated speed. The roof of the pulp chamber was removed using an EndoZ bur (Dentsply Maillefer), and the canal orifices were located using a DG-16 explorer (Hu-Friedy, Chicago, IL, USA). The length of each of the three root canals was determined radiographically by placing the #15 K-file (Dentsply Maillefer) into each canal. The working length (WL) was established as 1 mm short of the apex. 

The type of instrumentation for the particular tooth was chosen based on the randomization protocol. In Group 1, root canal instrumentations were carried out using the adaptive XP-endo Shaper (FKG Dentaire, La Chaux-de-Fonds, Switzerland); for Groups 2 and 3, Kedo-S pediatric rotary files (D1 and E1; Reeganz Dental Care Private Limited, Chennai, India) and a hand K-file (Dentsply Maillefer, Tulsa, OK, USA) up to #30 were used, respectively. 

The mechanized files were operated using the X-smart Plus endo-motor (Dentsply Maillefer). The setting for the XP-endo Shaper was 800 rpm and 1 Ncm torque, and for the Kedo-S files, a setting of 250 rpm and 2.2 Ncm torque was used. Between each file, irrigation was performed using 2.5% sodium hypochlorite (NaOCl; Prime Dental, Mumbai, India), and EDTA gel (RC Help; Prime Dental, Mumbai, India) was used as a lubricant. A final irrigation regime was performed utilizing 2 mL of 17% aqueous EDTA (DentWash; Prime Dental, Mumbai, India) and 4 mL of normal saline for all the groups.

#### 2.2.1. XP-endo Shaper Instrumentation

The allocated patients underwent root canal instrumentation using 21 mm XP-endo Shaper files. Following access cavity preparation, the canal patency was checked with a #10 K-file, and a glide path until #15 K-file was created if needed. The root canals were flooded with 2 mL of warm 2.5% NaOCl, and the XP-endo Shaper file was first placed passively until it met resistance. The tip was then retracted coronally, the endomotor was activated at a speed of 800 rpm and 1 Ncm torque, and the file was reintroduced. The file was used with light vertical strokes 4–5 times toward the WL. Once the WL was reached, the file was retracted and cleaned, apical patency was verified with the #15 K-file, the canal was irrigated again with 4 mL of warm NaOCl, and the file was used again for additional 15 strokes to the WL deeming completion of instrumentation, followed by irrigating the canal by 4 mL of warm NaOCl, according to manufacturer’s instruction. The final regime of irrigation with 17% EDTA and normal saline was performed, as mentioned earlier.

#### 2.2.2. Kedo-S Instrumentation

For the patients in this group, root canal instrumentation was carried out utilizing Kedo-S E1 files (16 mm), according to the manufacturer’s instructions. A tender in-and-out motion was used until the WL was reached. If the file met resistance before reaching the WL, it was retracted and cleaned, the canal was irrigated (2 mL of NaOCl), canal patency was confirmed (using a #15 K-file), and the file was reintroduced. Once the WL was reached, the file was retracted, and the was canal irrigated with 4 mL of NaOCl. The file was introduced in a vertical pecking motion until the WL was reached for an additional 5 strokes, deeming the completion of instrumentation, as stated by the manufacturer. The canals were irrigated again with 4 mL of NaOCl, and the final irrigation regime was performed.

#### 2.2.3. Hand Instrumentation

The root canal instrumentation for the participants in this group was performed using the quarter-turn-and-pull motion. Stainless steel K-files were maneuvered in a sequence of #15/0.02, #20/0.02, #25/0.02, and #30/0.02 hand K-files (Mani, Tokyo, Japan). Between each file, the canal was irrigated by 2 mL of NaOCl. After the last file, the canal was irrigated by an additional 2 mL of NaOCl, and the final irrigation regime was performed.

### 2.3. Root Canal Obturation

Post root canal instrumentation and final irrigation, the canals were dried with #30 paper points, a 0.04 taper for Groups 1 and 2, and a 0.02 taper for Group 3. The root canals were then filled with an iodoform-based calcium hydroxide paste (Metapex, META Biomed, Colmar, PA, USA), utilizing the pressure syringe with a needle. Furthermore, the hand file was used to push the paste to just short of the apex. The coronal surplus of the paste was excavated, and the coronal cavity was filled with glass ionomer cement (Vitrebond, 3M ESPE, St Paul, MN, USA). Finally, a preformed metallic crown (3M ESPE) was adapted and cemented (PCA, SS White, Gloucester, UK) in the same visit.

#### 2.3.1. Instrumentation Time 

The total instrumentation time was measured using a digital stopwatch. The time measurement started with the introduction of the first file into the first canal and stopped after the final saline irrigation. The corresponding instrumentation time was noted for each tooth by the operator. 

#### 2.3.2. Quality of Obturation

**Figure 3:**
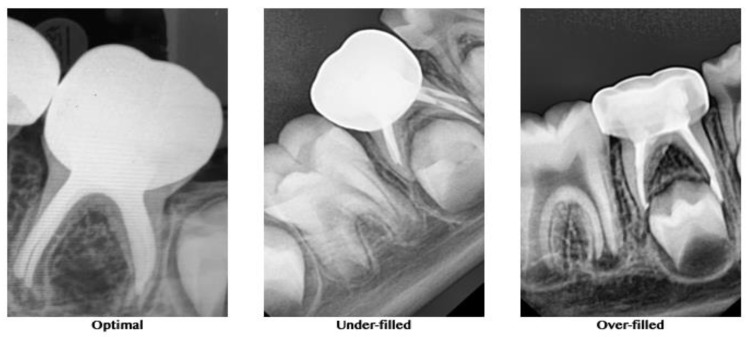
Representative radiographs for the quality of obturation.

A postobturation radiograph was taken for further evaluation and comparison of the quality of obturation between the groups. The quality of the root canal obturation was recorded, according to O’Riordan and Coll [23] as optimal (1 mm short of the apex), underfilled (2 mm short of the apex), or overfilled (beyond the apex) (Figure 3). 

### 2.4. Statistical Analysis

Data were tabulated, and analysis was performed using one-way ANOVA to compare the instrumentation times between the three groups, followed by the application of Tukey’s HSD test for a comparison between the groups. The quality of obturation of the 3 groups was assessed by application of a chi-square test. The SPSS v. 22 (IBM, Armonk, New York, NY, United States) statistical program was used to scrutinize the data.

## 3. Results

The demographic data of the three groups are presented in Table 1. As the time data followed a normal distribution (Kolmogorov–Smirnov and Shapiro–Wilks tests), statistical analysis was performed by the application of the parametric method. 

### 3.1. Time Requited for Instrumentation

The time required for the instrumentation of the primary teeth was 10.9 (±0.7), 14.8 (±1.2), and 19.9 (±1.0) min for the XP-endo Shaper, Kedo-S, and hand K-files, respectively. A significant difference was found between the groups (*p* < 0.0001; ANOVA). Tukey’s HSD test was further applied and indicated that XP-endo Shaper group was associated with a significantly smaller instrumentation time (*p* < 0.01), differentiating it from the other groups (Table 2).

### 3.2. Quality of Obturation

In the XP-endo Shaper group, 19 of the 25 teeth were optimally filled, 2 were underfilled, and 4 were overfilled. In the Kedo-S group, 15 of the 25 teeth were optimally filled, 3 were underfilled, and 7 were overfilled. Finally, in the hand K-file group, 12 of the 25 teeth, were optimally filled, 8 were underfilled, and 5 were overfilled. The results of the quality of obturation are presented in Table 3. The teeth treated with the XP-endo Shaper exhibited significantly better obturation results compared to the other groups (*p* < 0.01, Chi-square test).

## 4. Discussion

Pulpectomy is indicated for restorable, pulpally infected primary molars to preserve their function [4,5,8,9,12,13,17,23]. Meticulous chemomechanical preparation of the root canal and obturation with a resorbable biocompatible material until optimum length are essential to the success of a pulpectomy procedure [24].

Prolonged procedures may compromise the cooperation of the pediatric patient; therefore, there is a benefit in shortening the time required to perform the pulpectomy procedure, provided that the quality of the cleaning and obturation of the canals are not negatively affected. 

Traditional root canal instrumentation using K-files is a prolonged procedure associated with other potential drawbacks. Ledging, canal transportation, and zipping of the apical foramen are among the major mishaps when using this method. Mechanized rotary NiTi files were introduced to avoid such mishaps and also to shorten the time required for canal instrumentation [4,24]. 

In the present study, the time required to complete the instrumentation of all three canals of primary molars with rotary files was shorter by 20% compared to hand instrumentation with K-files. Also, the root canal instrumentation was carried out 1 mm short of the WL, hence no damage was caused to the underlying permanent tooth buds. These results of the present study are in agreement with those of Ochoa-Romero et al. [24], who also reported the significant shortening of instrumentation time when rotary files were used in primary molars. Silva et al. [7] reported much shorter instrumentation times with both rotary and hand files (3.5 and 9.1 min, respectively); however, their study was conducted in vitro, which may explain the difference from the present study results.

The use of the new XP-endo Shaper files was associated with reduced time to complete the instrumentation of the three canals: 10.9 (±0.7) min compared to 14.8 (±1.2) and 19.9 (±1.0) min with Kedo-S rotary files and K-files, respectively. No previous report on clinical instrumentation time with the XP-endo Shaper in primary molars was found to date.

The adaptive XP-endo Shaper has been reported to have a totally different mode of action than rotary files. NiTi rotary files have improved flexibility, but they have a fixed shape and taper and are thus likely to produce a space that represents their shape. This may be effective in narrow canals with a circular cross-section, but in irregularly shaped and ribbon-shaped root canals, rotary files have proven less satisfying [14,25,26]. In such canals, instrumentation with rotary files may result in a circular preparation, leaving uninstrumented buccal and/or lingual recesses. The XP-endo Shaper, on the other hand, is a flexible, snake-like file that is likely to form a space representing the “envelope of motion” of the rotating file. This envelope of motion is flexible and may contract and expand, as required in a given canal, thus the XP-endo Shaper file may adapt itself to oval- and even ribbon-shaped canals [19,20,27], such as those often found in primary molars [28].

Evaluating the quality of obturation in the present study was limited to the method proposed by O’Riordan and Coll [22] and used the criteria of optimal (1 mm short of the apex), underfilled (2 mm short of the apex), or overfilled (beyond the apex). Obturation in the current study was carried out using Metapex, an iodoform-based calcium hydroxide cement. This cement possesses better resorbing ability and disinfectant properties compared to conventional zinc oxide eugenol cement. Additionally, it is resorbed by macrophages faster than the primary root [29]. This cement is advantageous, exhibiting no foreign body reaction when extruded into furcal or apical areas when used for obturation. There are no reports of any effect of the extruded Metapex on permanent tooth buds to date. It has also been observed that the extruded cement usually resorbs within 1–2 weeks [30]. The present finding that root canals instrumented with the XP-endo Shaper allowed a more frequent (76%) optimal obturation result, according to the above criteria, may be explained by previous ex vivo studies of this adaptive file. The ability of this file to adequately instrument and clean the canals of permanent teeth with an oval cross-section has been studied using microCT [20,27,31,32]. It has been demonstrated that the XP-endo Shaper resulted in better 3D instrumentation than rotary files, affecting a higher percentage of the root canal walls [20,27,31,32]. It could be that a cleaner canal, without debris remnants in its recesses, is easier to obturate with the method used in the present study.

The preservation of primary dentition is indispensable for children’s oral and general health [33]. In addition, they act as innate space maintainers for permanent dentition [34]. They aid in mastication, the preservation of arch length, the prevention of abnormal tongue movements, and phonetics. Hence, salvaging a natural primary tooth, if possible, should always be of prime importance.

A limitation of the present study was that the quality of obturation was assessed only from 2D periapical radiographs. In a clinical study in children, this was the only possibility. It will be of interest to conduct an ex vivo study on extracted primary molars, similar to the present study, using microCT to evaluate the quality of obturation allowed by the three instrumentation methods, in a real, 3D manner. Nevertheless, such an investigation was beyond the scope of the present clinical trial. 

## 5. Conclusions

The use of adaptive XP-endo Shaper instrumentation resulted in faster instrumentation and better obturation quality compared to pediatric rotary files and manual instrumentation.

## Figures and Tables

**Table 1:** Demographic data and teeth enrolled in per group of treatment for the 3 groups.

Group of Treatment	Sample Size	Age Median (Y)	Gender Male/Female	Number of Teeth Treated for Pulpectomy #54-55-64-65-74-75-84-85
XP-endo Shaper	25	5.7	16/09	4-3-4-4-4-1-4-1
Kedo-S	25	5.6	10/15	4-3-4-4-4-1-4-1
Hand K-files	25	5.9	07/18	4-3-4-4-4-1-4-1

**Table 2:** Instrumentation time (minutes).

Files	Sample Size	Mean (±Standard Deviation)	Tukey HSD Test
XP-Endo Shaper (XPS)	25	10.9 (±0.7) ^a^	<KS and HF
Kedo-S (KS)	25	14.8 (±1.2) ^b^	>XPS, <HF
Hand K-files (HF)	25	19.9 (±1.0) ^c^	>XPS and KS

Different superscript lower-case alphabets present significant differences after the application of one-way ANOVA (*p* < 0.0001). Superscript a, b, and c represent instrumentation time is ascending order from lowest to the highest time required.

**Table 3:** Quality of obturation.

Files	Sample Size	Optimal	Underfilled	Overfilled
XP-endo Shaper (XPS)	25	19 (76%)	2 (8%)	4 (16%)
Kedo-S (KS)	25	15 (60%)	3 (12%)	7 (28%)
Hand K-files (HF)	25	12 (48%)	8 (32%)	5 (20%)

## Data Availability

The current manuscript has been registered with the Clinical Trial Registry of India (CTRI/2019/12/022370) and no other data sharing is not applicable to this article.
